# Supplementary material for: A Likelihood Approach for Real-Time Calibration of Stochastic Compartmental Epidemic Models
Source: PLoS Comput Biol. 2017 Jan 17;13(1):e1005257. doi: 10.1371/journal.pcbi.1005257 (PMC5240920; doi:10.1371/journal.pcbi.1005257)
Supplement: S1 File — (TAR.GZ) [file pcbi.1005257.s014.tar.gz › HSPH_Online-SI-Revision/PF08/n1-severe/n1-severe_PF2_table-4.pdf]

| | | | | | | | | | | | | | | | | | | | | | | | | | | | | | | |
|---|---|---|---|---|---|---|---|---|---|---|---|---|---|---|---|---|---|---|---|---|---|---|---|---|---|---|---|---|---|---|
| 1 | newly 3 weeks spec | 33. | 33.65 | {12., 59.} | 46.3333 | 0.220018 | newly 3 weeks cumul | 81. | 107.39 | {40., 209.} | 59.7901 | 0.213777 | newly 5 weeks spec | 34. | 27.25 | {9., 46.} | 40.2059 | 0.24297 | newly 5 weeks cumul | 158. | 166.05 | {67., 311.} | 42.6013 | 0.190957 | newly 58 weeks cumul | 531. | 383.32 | {135., 622.} | 35.9661 | 0.234624 |
| 2 | newly 3 weeks spec | 55. | 48.65 | {15., 93.} | 47.5818 | 0.26922 | newly 3 weeks cumul | 130. | 145.85 | {48., 265.} | 54.2538 | 0.235572 | newly 5 weeks spec | 53. | 43.6 | {12., 74.} | 45.8491 | 0.320373 | newly 5 weeks cumul | 247. | 236.44 | {78., 435.} | 46.8502 | 0.242295 | newly 60 weeks cumul | 592. | 475.63 | {178., 768.} | 33.9645 | 0.209026 |
| 3 | newly 3 weeks spec | 32. | 40.16 | {13., 76.} | 58.875 | 0.231389 | newly 3 weeks cumul | 74. | 126.83 | {46., 237.} | 89.0676 | 0.258341 | newly 5 weeks spec | 43. | 33.65 | {10., 60.} | 42.9535 | 0.269365 | newly 5 weeks cumul | 164. | 197.92 | {69., 348.} | 52.8537 | 0.210073 | newly 59 weeks cumul | 553. | 417.52 | {137., 640.} | 34.9837 | 0.221167 |
| 4 | newly 3 weeks spec | 58. | 40.74 | {3., 76.} | 47.7931 | 0.475054 | newly 3 weeks cumul | 144. | 120.79 | {7., 231.} | 52.1319 | 0.459623 | newly 5 weeks spec | 59. | 37.84 | {3., 77.} | 51.8644 | 0.524147 | newly 5 weeks cumul | 286. | 198.04 | {16., 383.} | 45.3217 | 0.477379 | newly 62 weeks cumul | 620. | 460.2 | {73., 796.} | 39.271 | 0.415892 |
| 5 | newly 3 weeks spec | 56. | 36.63 | {1., 79.} | 54.4464 | 0.612168 | newly 3 weeks cumul | 140. | 111.45 | {5., 234.} | 54.95 | 0.467624 | newly 5 weeks spec | 40. | 31.55 | {0., 72.} | 62.125 | 0.643947 | newly 5 weeks cumul | 241. | 178.3 | {8., 356.} | 53.0622 | 0.492116 | newly 61 weeks cumul | 534. | 370.47 | {17., 726.} | 46.4213 | 0.48779 |
| 6 | newly 3 weeks spec | 31. | 38.92 | {13., 71.} | 60.2581 | 0.26989 | newly 3 weeks cumul | 71. | 119.44 | {42., 211.} | 87.1549 | 0.264822 | newly 5 weeks spec | 40. | 31.77 | {9., 59.} | 45.625 | 0.328099 | newly 5 weeks cumul | 159. | 187.9 | {74., 355.} | 54.0881 | 0.230354 | newly 58 weeks cumul | 620. | 397.76 | {126., 653.} | 40.229 | 0.293346 |
| 7 | newly 3 weeks spec | 37. | 40.19 | {16., 76.} | 49.4865 | 0.218939 | newly 3 weeks cumul | 101. | 128. | {54., 250.} | 56.0198 | 0.206709 | newly 5 weeks spec | 30. | 32.72 | {11., 62.} | 51.9333 | 0.23737 | newly 5 weeks cumul | 170. | 198.3 | {87., 368.} | 49.6941 | 0.198635 | newly 58 weeks cumul | 392. | 406.51 | {132., 651.} | 38.0179 | 0.186872 |
| 8 | newly 3 weeks spec | 83. | 38.84 | {3., 78.} | 55.4699 | 0.60769 | newly 3 weeks cumul | 181. | 117.23 | {22., 243.} | 51.3315 | 0.490032 | newly 5 weeks spec | 97. | 34.39 | {2., 65.} | 66.299 | 0.76924 | newly 5 weeks cumul | 384. | 188.92 | {25., 367.} | 52.8438 | 0.570555 | newly 62 weeks cumul | 588. | 399.5 | {44., 736.} | 44.1769 | 0.474786 |
| 9 | newly 3 weeks spec | 38. | 35.87 | {15., 61.} | 43.8158 | 0.217697 | newly 3 weeks cumul | 79. | 114.44 | {48., 210.} | 64.3797 | 0.202217 | newly 5 weeks spec | 31. | 30.02 | {10., 57.} | 48.7097 | 0.269213 | newly 5 weeks cumul | 150. | 176.73 | {82., 311.} | 48.98 | 0.189614 | newly 58 weeks cumul | 454. | 386.91 | {149., 670.} | 37.1388 | 0.220346 |
| 10 | newly 3 weeks spec | 67. | 43.99 | {16., 81.} | 45.2687 | 0.304125 | newly 3 weeks cumul | 156. | 144.42 | {52., 269.} | 43.7308 | 0.224917 | newly 5 weeks spec | 47. | 35.79 | {7., 66.} | 44.1915 | 0.331206 | newly 5 weeks cumul | 273. | 220.46 | {76., 394.} | 41.9853 | 0.243907 | newly 58 weeks cumul | 540. | 415.46 | {129., 661.} | 35.6481 | 0.229718 |
| 11 | newly 3 weeks spec | 25. | 26.54 | {7., 52.} | 51.6 | 0.263021 | newly 3 weeks cumul | 76. | 88.11 | {36., 158.} | 51.0132 | 0.213594 | newly 5 weeks spec | 31. | 19.99 | {4., 39.} | 50.9355 | 0.437853 | newly 5 weeks cumul | 146. | 131.46 | {47., 238.} | 43.8904 | 0.239225 | newly 55 weeks cumul | 505. | 257.48 | {57., 441.} | 50.8673 | 0.408743 |
| 12 | newly 3 weeks spec | 95. | 43.5 | {6., 90.} | 57.0105 | 0.6219 | newly 3 weeks cumul | 232. | 132.4 | {16., 272.} | 52.6724 | 0.486381 | newly 5 weeks spec | 59. | 37.65 | {5., 76.} | 50.7966 | 0.489047 | newly 5 weeks cumul | 391. | 210.58 | {35., 406.} | 50.491 | 0.475822 | newly 63 weeks cumul | 569. | 442.07 | {87., 750.} | 37.6397 | 0.311531 |
| 13 | newly 3 weeks spec | 46. | 39.72 | {15., 71.} | 47.2609 | 0.272281 | newly 3 weeks cumul | 111. | 124.64 | {56., 219.} | 47.0991 | 0.193183 | newly 5 weeks spec | 49. | 31.72 | {8., 62.} | 49.6735 | 0.365845 | newly 5 weeks cumul | 221. | 193.69 | {74., 344.} | 42.9638 | 0.224566 | newly 58 weeks cumul | 585. | 390.04 | {146., 657.} | 40.7487 | 0.276115 |
| 14 | newly 3 weeks spec | 59. | 40.23 | {3., 87.} | 53.9153 | 0.516635 | newly 3 weeks cumul | 122. | 127.66 | {11., 259.} | 67. | 0.417577 | newly 5 weeks spec | 75. | 35.5 | {3., 73.} | 54.88 | 0.627337 | newly 5 weeks cumul | 272. | 202.67 | {25., 405.} | 51.9228 | 0.438936 | newly 63 weeks cumul | 614. | 430.48 | {58., 761.} | 38.9642 | 0.37027 |
| 15 | newly 3 weeks spec | 43. | 37.25 | {12., 65.} | 46.907 | 0.265077 | newly 3 weeks cumul | 132. | 117.35 | {42., 212.} | 41.6288 | 0.214314 | newly 5 weeks spec | 36. | 28.88 | {6., 57.} | 48.2222 | 0.339526 | newly 5 weeks cumul | 229. | 178.27 | {62., 325.} | 43.0087 | 0.251688 | newly 57 weeks cumul | 373. | 343.02 | {95., 606.} | 43.6247 | 0.242613 |
| 16 | newly 3 weeks spec | 37. | 41.06 | {2., 87.} | 72.7568 | 0.481606 | newly 3 weeks cumul | 113. | 130.54 | {11., 263.} | 74.3363 | 0.422631 | newly 5 weeks spec | 55. | 36.38 | {2., 74.} | 51.3455 | 0.551647 | newly 5 weeks cumul | 226. | 207.5 | {25., 410.} | 57.8584 | 0.422152 | newly 63 weeks cumul | 577. | 436.14 | {44., 743.} | 37.6672 | 0.35542 |
| 17 | newly 3 weeks spec | 80. | 43.59 | {4., 88.} | 53.5125 | 0.582094 | newly 3 weeks cumul | 176. | 131.76 | {16., 268.} | 54.2045 | 0.44045 | newly 5 weeks spec | 63. | 36.8 | {2., 76.} | 52.2857 | 0.577868 | newly 5 weeks cumul | 335. | 208.86 | {28., 408.} | 49.1582 | 0.45759 | newly 63 weeks cumul | 583. | 428.4 | {58., 750.} | 39.4237 | 0.366621 |
| 18 | newly 3 weeks spec | 27. | 45.6 | {3., 90.} | 112.296 | 0.484325 | newly 3 weeks cumul | 67. | 142. | {18., 289.} | 146.507 | 0.454279 | newly 5 weeks spec | 23. | 36.71 | {2., 78.} | 110.826 | 0.515129 | newly 5 weeks cumul | 126. | 221.49 | {31., 425.} | 114.357 | 0.426597 | newly 63 weeks cumul | 593. | 435.49 | {77., 743.} | 38.2411 | 0.337978 |
| 19 | newly 3 weeks spec | 46. | 31.86 | {9., 63.} | 45.8696 | 0.328396 | newly 3 weeks cumul | 143. | 106.95 | {37., 205.} | 44.0769 | 0.26552 | newly 5 weeks spec | 45. | 25.17 | {5., 45.} | 49.9778 | 0.428815 | newly 5 weeks cumul | 247. | 160.9 | {55., 315.} | 45.8785 | 0.305913 | newly 56 weeks cumul | 533. | 329.01 | {106., 533.} | 41.9944 | 0.314129 |
| 20 | newly 3 weeks spec | 63. | 31.76 | {8., 63.} | 55.3333 | 0.443662 | newly 3 weeks cumul | 168. | 104.54 | {38., 206.} | 50.6905 | 0.334866 | newly 5 weeks spec | 55. | 23.41 | {3., 44.} | 59.2909 | 0.565786 | newly 5 weeks cumul | 291. | 155.17 | {54., 299.} | 53.1443 | 0.385728 | newly 56 weeks cumul | 494. | 297.32 | {68., 509.} | 44.1296 | 0.340499 |
| 21 | newly 3 weeks spec | 38. | 44.3 | {5., 91.} | 72.7895 | 0.435601 | newly 3 weeks cumul | 97. | 139.73 | {19., 266.} | 90.732 | 0.406166 | newly 5 weeks spec | 50. | 38.48 | {2., 78.} | 53.76 | 0.512779 | newly 5 weeks cumul | 199. | 219.77 | {32., 409.} | 63.9045 | 0.377058 | newly 63 weeks cumul | 538. | 443.24 | {87., 758.} | 39.119 | 0.312937 |
| 22 | newly 3 weeks spec | 77. | 36.57 | {11., 69.} | 57.5455 | 0.46496 | newly 3 weeks cumul | 210. | 118.27 | {45., 247.} | 53.2619 | 0.368358 | newly 5 weeks spec | 61. | 29.3 | {8., 55.} | 53.8361 | 0.457446 | newly 5 weeks cumul | 359. | 180.64 | {65., 347.} | 54.4847 | 0.401328 | newly 57 weeks cumul | 537. | 360.24 | {99., 610.} | 40.4618 | 0.288548 |
| 23 | newly 3 weeks spec | 30. | 27.07 | {2., 60.} | 60.5 | 0.448074 | newly 3 weeks cumul | 92. | 87.68 | {10., 170.} | 60.413 | 0.385183 | newly 5 weeks spec | 37. | 20.5 | {0., 47.} | 58.8108 | 0.640257 | newly 5 weeks cumul | 174. | 132.24 | {12., 269.} | 54.2299 | 0.402746 | newly 59 weeks cumul | 461. | 295.35 | {33., 584.} | 50.4447 | 0.429724 |
| 24 | newly 3 weeks spec | 38. | 16.24 | {0., 36.} | 60.8421 | 0.693148 | newly 3 weeks cumul | 121. | 56.04 | {5., 120.} | 57.7521 | 0.587363 | newly 5 weeks spec | 32. | 13.2 | {0., 35.} | 64.4375 | 0.779553 | newly 5 weeks cumul | 201. | 83.71 | {8., 186.} | 60.6119 | 0.643692 | newly 56 weeks cumul | 407. | 177.44 | {8., 384.} | 63.8034 | 0.715977 |
| 25 | newly 3 weeks spec | 55. | 23.12 | {4., 45.} | 60.6182 | 0.594731 | newly 3 weeks cumul | 180. | 76.14 | {16., 159.} | 59.8556 | 0.52592 | newly 5 weeks spec | 62. | 17.45 | {1., 31.} | 71.8548 | 0.82372 | newly 5 weeks cumul | 314. | 113.46 | {26., 227.} | 64.707 | 0.601149 | newly 54 weeks cumul | 547. | 229.06 | {31., 431.} | 59.9707 | 0.590466 |
| 26 | newly 3 weeks spec | 41. | 38.15 | {3., 79.} | 57.3415 | 0.476957 | newly 3 weeks cumul | 123. | 114.53 | {5., 224.} | 56.0407 | 0.411586 | newly 5 weeks spec | 42. | 34.47 | {0., 73.} | 60.881 | 0.602733 | newly 5 weeks cumul | 210. | 186.65 | {16., 356.} | 54.4143 | 0.428892 | newly 61 weeks cumul | 500. | 409.69 | {22., 752.} | 43.834 | 0.407013 |
| 27 | newly 3 weeks spec | 55. | 29.08 | {1., 66.} | 56.1818 | 0.59745 | newly 3 weeks cumul | 170. | 92.3 | {9., 183.} | 53.0235 | 0.516452 | newly 5 weeks spec | 64. | 24.14 | {1., 53.} | 65.3125 | 0.753145 | newly 5 weeks cumul | 324. | 142.74 | {15., 292.} | 58.3272 | 0.578976 | newly 60 weeks cumul | 489. | 313.44 | {38., 606.} | 49.3579 | 0.431576 |
| 28 | newly 3 weeks spec | 52. | 41.87 | {2., 87.} | 56.0962 | 0.506728 | newly 3 weeks cumul | 108. | 131.45 | {15., 263.} | 77.9722 | 0.419418 | newly 5 weeks spec | 42. | 37.3 | {2., 77.} | 58.1429 | 0.513218 | newly 5 weeks cumul | 207. | 210.23 | {25., 409.} | 62.285 | 0.414512 | newly 63 weeks cumul | 611. | 433.99 | {44., 761.} | 38.3191 | 0.369319 |
| 29 | newly 3 weeks spec | 39. | 29.58 | {8., 63.} | 49.5897 | 0.328268 | newly 3 weeks cumul | 112. | 98.28 | {37., 164.} | 45.1429 | 0.24273 | newly 5 weeks spec | 44. | 23.59 | {3., 48.} | 54.2955 | 0.489857 | newly 5 weeks cumul | 200. | 148.6 | {53., 283.} | 46.17 | 0.28964 | newly 56 weeks cumul | 440. | 299.36 | {59., 548.} | 44.5136 | 0.336503 |
| 30 | newly 3 weeks spec | 46. | 36.78 | {11., 69.} | 44.5217 | 0.292132 | newly 3 weeks cumul | 145. | 118.16 | {42., 233.} | 43.6552 | 0.252289 | newly 5 weeks spec | 43. | 29.22 | {6., 56.} | 46.7442 | 0.385013 | newly 5 weeks cumul | 245. | 180.88 | {65., 340.} | 43.4857 | 0.274735 | newly 57 weeks cumul | 463. | 350.03 | {87., 566.} | 38.1361 | 0.264741 |
| 31 | newly 3 weeks spec | 17. | 37.8 | {11., 64.} | 135.059 | 0.338001 | newly 3 weeks cumul | 48. | 120.33 | {41., 210.} | 156.688 | 0.35854 | newly 5 weeks spec | 16. | 31.76 | {11., 57.} | 112.75 | 0.315435 | newly 5 weeks cumul | 108. | 187.01 | {65., 318.} | 88.1759 | 0.258222 | newly 58 weeks cumul | 420. | 397.15 | {171., 638.} | 37.931 | 0.203832 |
| 32 | newly 3 weeks spec | 35. | 44.66 | {11., 94.} | 70.5714 | 0.296352 | newly 3 weeks cumul | 65. | 137.82 | {47., 250.} | 125.354 | 0.31027 | newly 5 weeks spec | 21. | 36.97 | {10., 72.} | 102.524 | 0.351028 | newly 5 weeks cumul | 124. | 215.36 | {71., 433.} | 94.2742 | 0.27282 | newly 60 weeks cumul | 460. | 447.98 | {155., 725.} | 35.8652 | 0.191818 |
| 33 | newly 3 weeks spec | 48. | 41.49 | {4., 90.} | 61.8542 | 0.520437 | newly 3 weeks cumul | 140. | 129.92 | {15., 268.} | 63.5429 | 0.437017 | newly 5 weeks spec | 62. | 36.63 | {2., 77.} | 54.0484 | 0.584458 | newly 5 weeks cumul | 272. | 205.58 | {27., 408.} | 52.3897 | 0.434102 | newly 63 weeks cumul | 628. | 438.46 | {60., 752.} | 38.5318 | 0.351297 |
| 34 | newly 3 weeks spec | 16. | 24.58 | {9., 50.} | 77. | 0.28669 | newly 3 weeks cumul | 68. | 80.83 | {31., 139.} | 49.9265 | 0.202039 | newly 5 weeks spec | 22. | 19.54 | {3., 37.} | 49.2727 | 0.371552 | newly 5 weeks cumul | 123. | 120.95 | {39., 207.} | 42.3333 | 0.21332 | newly 55 weeks cumul | 356. | 253.29 | {55., 456.} | 46.1376 | 0.330045 |
| 35 | newly 3 weeks spec | 73. | 40.39 | {3., 87.} | 54.0959 | 0.560641 | newly 3 weeks cumul | 170. | 129.9 | {15., 259.} | 55.8706 | 0.451525 | newly 5 weeks spec | 62. | 35.64 | {2., 77.} | 53.5484 | 0.608825 | newly 5 weeks cumul | 317. | 205.28 | {25., 405.} | 50.1956 | 0.462166 | newly 63 weeks cumul | 588. | 435.93 | {70., 763.} | 39.2024 | 0.363335 |
| 36 | newly 3 weeks spec | 88. | 53.78 | {6., 100.} | 48.0455 | 0.458939 | newly 3 weeks cumul | 210. | 164.75 | {15., 316.} | 46.2905 | 0.400307 | newly 5 weeks spec | 57. | 42.52 | {3., 85.} | 51.0526 | 0.462034 | newly 5 weeks cumul | 377. | 255.94 | {25., 454.} | 42.7586 | 0.406748 | newly 64 weeks cumul | 488. | 481.64 | {109., 803.} | 40.7787 | 0.308468 |
| 37 | newly 3 weeks spec | 41. | 23.55 | {6., 41.} | 49.2439 | 0.40304 | newly 3 weeks cumul | 138. | 79.56 | {29., 138.} | 49.7681 | 0.363165 | newly 5 weeks spec | 36. | 18.71 | {1., 37.} | 54.6944 | 0.585997 | newly 5 weeks cumul | 224. | 119.06 | {35., 215.} | 51.9196 | 0.402497 | newly 54 weeks cumul | 482. | 241.61 | {36., 426.} | 52.3714 | 0.46212 |
| 38 | newly 3 weeks spec | 82. | 44.18 | {8., 84.} | 49.6585 | 0.512413 | newly 3 weeks cumul | 203. | 133.74 | {23., 252.} | 47.33 | 0.444289 | newly 5 weeks spec | 63. | 36.79 | {2., 72.} | 52.0159 | 0.537842 | newly 5 weeks cumul | 360. | 212.06 | {26., 389.} | 45.3944 | 0.464337 | newly 62 weeks cumul | 525. | 420.15 | {73., 736.} | 40.299 | 0.380599 |
| 39 | newly 3 weeks spec | 46. | 39.28 | {9., 76.} | 48.3043 | 0.296281 | newly 3 weeks cumul | 116. | 129.78 | {42., 256.} | 54.1897 | 0.234474 | newly 5 weeks spec | 36. | 31.77 | {8., 58.} | 50.3611 | 0.328878 | newly 5 weeks cumul | 202. | 196.61 | {64., 383.} | 47.1535 | 0.238014 | newly 58 weeks cumul | 447. | 377.62 | {133., 631.} | 39.0694 | 0.242041 |
| 40 | newly 3 weeks spec | 52. | 48.02 | {3., 103.} | 61.6538 | 0.459401 | newly 3 weeks cumul | 151. | 136.08 | {9., 284.} | 59.2053 | 0.446779 | newly 5 weeks spec | 41. | 40.68 | {3., 84.} | 59.8537 | 0.42261 | newly 5 weeks cumul | 250. | 221.34 | {17., 433.} | 55.424 | 0.424134 | newly 64 weeks cumul | 483. | 496.25 | {115., 783.} | 42.1718 | 0.314123 |
| 41 | newly 3 weeks spec | 64. | 38.01 | {2., 79.} | 52.6094 | 0.588615 | newly 3 weeks cumul | 223. | 115.15 | {5., 234.} | 53.7803 | 0.551647 | newly 5 weeks spec | 76. | 32.57 | {0., 72.} | 61.5921 | 0.752209 | newly 5 weeks cumul | 393. | 184.1 | {8., 356.} | 55.0331 | 0.587938 | newly 61 weeks cumul | 574. | 382.83 | {18., 726.} | 44.716 | 0.462526 |
| 42 | newly 3 weeks spec | 30. | 38.23 | {2., 79.} | 80.1667 | 0.497108 | newly 3 weeks cumul | 92. | 116.83 | {5., 234.} | 77.1848 | 0.419608 | newly 5 weeks spec | 39. | 32.45 | {0., 71.} | 57.2564 | 0.543598 | newly 5 weeks cumul | 182. | 185.55 | {12., 353.} | 60.0165 | 0.418684 | newly 61 weeks cumul | 534. | 397.47 | {24., 738.} | 42.0318 | 0.403867 |
| 43 | newly 3 weeks spec | 35. | 23.4 | {3., 49.} | 52.9143 | 0.466749 | newly 3 weeks cumul | 108. | 80.28 | {18., 165.} | 49.0185 | 0.362178 | newly 5 weeks spec | 36. | 18.27 | {0., 37.} | 57.5278 | 0.630713 | newly 5 weeks cumul | 191. | 119.94 | {22., 237.} | 51.0366 | 0.4264 | newly 54 weeks cumul | 401. | 226.39 | {31., 426.} | 52.0274 | 0.489047 |
| 44 | newly 3 weeks spec | 64. | 39.41 | {3., 76.} | 49.3281 | 0.477292 | newly 3 weeks cumul | 144. | 118.49 | {16., 232.} | 51.5486 | 0.436331 | newly 5 weeks spec | 60. | 35.09 | {2., 66.} | 52.3833 | 0.562924 | newly 5 weeks cumul | 278. | 191.34 | {25., 356.} | 46.2086 | 0.457738 | newly 62 weeks cumul | 520. | 424.14 | {58., 752.} | 42.7538 | 0.402475 |
| 45 | newly 3 weeks spec | 36. | 28.88 | {1., 64.} | 58.7222 | 0.537047 | newly 3 weeks cumul | 140. | 92.91 | {7., 190.} | 50.75 | 0.474722 | newly 5 weeks spec | 47. | 22.35 | {0., 50.} | 60.617 | 0.748505 | newly 5 weeks cumul | 246. | 141.02 | {10., 306.} | 52.8618 | 0.51519 | newly 59 weeks cumul | 529. | 290.38 | {24., 553.} | 50.4499 | 0.50316 |
| 46 | newly 3 weeks spec | 25. | 37.99 | {14., 66.} | 73.8 | 0.26319 | newly 3 weeks cumul | 61. | 118.93 | {44., 208.} | 104.377 | 0.281219 | newly 5 weeks spec | 28. | 32.1 | {8., 58.} | 54.7857 | 0.288692 | newly 5 weeks cumul | 125. | 187.21 | {72., 323.} | 68.424 | 0.226432 | newly 58 weeks cumul | 517. | 403.58 | {139., 675.} | 37.0484 | 0.238524 |
| 47 | newly 3 weeks spec | 97. | 51. | {3., 105.} | 55.8969 | 0.577518 | newly 3 weeks cumul | 190. | 146.56 | {14., 305.} | 56.2211 | 0.453029 | newly 5 weeks spec | 95. | 42.42 | {3., 87.} | 56.2526 | 0.605615 | newly 5 weeks cumul | 400. | 236.23 | {28., 461.} | 50.0475 | 0.479668 | newly 64 weeks cumul | 577. | 469.1 | {61., 794.} | 39.1681 | 0.341073 |
| 48 | newly 3 weeks spec | 31. | 24.17 | {0., 53.} | 58.9355 | 0.582649 | newly 3 weeks cumul | 100. | 79. | {7., 169.} | 59.16 | 0.481872 | newly 5 weeks spec | 36. | 19.58 | {0., 46.} | 60.7778 | 0.6974 | newly 5 weeks cumul | 184. | 120.56 | {9., 269.} | 55.3696 | 0.516203 | newly 58 weeks cumul | 364. | 257.95 | {19., 531.} | 56.3819 | 0.54196 |
| 49 | newly 3 weeks spec | 70. | 42.45 | {8., 82.} | 48.1 | 0.461258 | newly 3 weeks cumul | 184. | 128.41 | {25., 252.} | 47.8424 | 0.4234 | newly 5 weeks spec | 55. | 35.97 | {3., 69.} | 51.8727 | 0.501905 | newly 5 weeks cumul | 319. | 203.4 | {30., 383.} | 45.7304 | 0.439331 | newly 62 weeks cumul | 565. | 407.28 | {73., 722.} | 41.377 | 0.398735 |
| 50 | newly 3 weeks spec | 37. | 44.95 | {11., 92.} | 66.1892 | 0.284977 | newly 3 weeks cumul | 82. | 134.06 | {42., 245.} | 90. | 0.274751 | newly 5 weeks spec | 52. | 37.91 | {9., 77.} | 49.6731 | 0.365514 | newly 5 weeks cumul | 186. | 215.35 | {64., 413.} | 57.6183 | 0.246661 | newly 60 weeks cumul | 449. | 452.59 | {178., 731.} | 34.51 | 0.185318 |
